# Mobile Messaging as Surveillance Tool during Pandemic (H1N1) 2009, Mexico

**DOI:** 10.3201/eid1609.100671

**Published:** 2010-09

**Authors:** Martín Lajous, Leon Danon, Ruy López-Ridaura, Christina M. Astley, Joel C. Miller, Scott F. Dowell, Justin J. O’Hagan, Edward Goldstein, Marc Lipsitch

**Affiliations:** Author affiliations: Center for Population Health Research National Institute of Public Health, Cuernavaca, Mexico (M. Lajous, R. López-Ridaura);; Harvard School of Public Health, Boston, Massachusetts, USA (M. Lajous, L. Danon, C.M. Astley, J.C. Miller, J.J. O’Hagan, E. Goldstein, M. Lipsitch);; University of Warwick, Coventry, UK (L. Danon);; National Institutes of Health, Bethesda, Maryland, USA (J.C. Miller);; Centers for Disease Control and Prevention, Atlanta, Georgia, USA (S.F. Dowell)

**Keywords:** Influenza, cell phone, surveillance, mobile messaging, survey, viruses, Mexico, letter

**To the Editor:** Pandemic (H1N1) 2009 highlighted challenges faced by disease surveillance systems. New approaches to complement traditional surveillance are needed, and new technologies provide new opportunities. We evaluated cell phone technology for surveillance of influenza outbreaks during the outbreak of pandemic (H1N1) 2009 in Mexico.

On May 12, 2009, at 2:20 pm, a random sample of 982,708 telephones from an 18 million nationwide network of mostly prepaid cell phones (*1*) received a text message invitation to a Ministry of Health survey. Influenza-like illness (ILI) in April, date of fever onset, severity, number of household members with ILI, age, influenza vaccination, household size, and number of children in each household were assessed (Technical Appendix Figure 1). ILI was defined as fever and cough or sore throat, and severe ILI was defined as inability to work, study, or maintain family care. Unstructured supplementary service data, an interactive platform available on most cell phones, was used. We obtained daily counts of suspected and confirmed cases of pandemic (H1N1) 2009 from the nationwide clinic-based surveillance system Sistema Nacional de Vigilancia Epidemiológica (SINAVE) (*2**,**3*).

Of 70,856 responses received, 56,551 (78.1%) were unique mobile numbers (5.8% response rate; only the first response was used). Within 3 hours, 53% of responses were received and by 24 hours, 89% were received. Mean (SD) age of respondents was 25.2 (10.4) years (Technical Appendix Table ). A total of 9,333 persons reported ILI and 49.3% had severe symptoms. Mean number of other persons with ILI in the household was 1.6 among respondents reporting severe disease and 0.3 among those with nonsevere disease (p<0.0001, by *t* test).

Epidemic curves for disease onset for confirmed and suspected cases of pandemic (H1N1) 2009 from SINAVE and daily proportion of severe cases from the cell phone survey are shown in the Figure. Daily counts of ILI were clustered around multiples of 5, and no distinct pattern was observed (Technical Appendix Figure 2). Use of the daily proportion of severe cases may partially correct for clustering and artifactual peaks by standardizing by total number of cases. The proportion of severe cases increased throughout the month beginning on April 1 (36.4%) and peaking on April 26 (57.9%). Two distinct decreases in severity of disease coincided with Semana Santa school vacation and school closures on April 24. These decreases are consistent with the decrease in the SINAVE epidemic curve.

**Figure:**
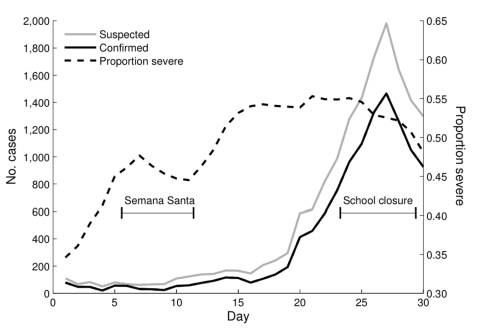
Proportion of severe cases of influenza-like illness (ILI) in Mexico, April 2009, from unstructured supplementary service data survey and confirmed and suspected cases of pandemic (H1N1) 2009 from Sistema Nacional de Vigilancia Epidemiológica. Suspected cases of pandemic (H1N1) 2009 are ILI cases for which no laboratory confirmation was possible. The daily proportion of reported severe cases and daily counts of confirmed and suspected cases of pandemic (H1N1) 2009 were smoothed by using a 5-day moving average.

The pattern of change in the proportion of severe ILI may be consistent with a decrease in transmission after control measures were implemented. The low response rate (5.8%) made it likely that respondents were not representative of the total population. Therefore, we did not make estimates of disease incidence. We were unable to determine whether a pathogen for which susceptibility was higher was responsible for the difference in number of ILI cases within the household of those reporting severe disease or whether respondents in households with several affected persons were more likely to report severe disease (Technical Appendix Table ). We observed unexpected peaks and a clustering of date of fever onset. However, the peak on April 1 may reflect disease at the end of March, and the decrease in daily proportion of severe cases may indicate lower incidence of ILI after school closures. Comparison of these data with epidemic curves for pandemic (H1N1) 2009 showed less variability than expected; no geographic variation was detected.

Our study was limited by potential selection bias, recall bias, and inclusion of mostly young persons from urban areas. Comparisons between reported cases and noncases are invalid because of the low response rate. However, comparisons within cases may be less prone to bias if they are more likely to respond.

Persons had difficulty remembering the exact date of fever onset. In 2 telephone surveys in New York City outside the influenza season (March and October–November 2003), a total of 20.8% and 19.6% of respondents, respectively, reported ILI within the past month (*4*), which were more than the rate of 12% during the peak of pandemic (H1N1) 2009 in New York (*5*). Use of daily proportion of severe cases may have partially corrected for this recall error. Also, persons may be more likely to report ILI if the date of onset was closer to the date of the survey. Nevertheless, a lower number of cases by the end of the month indicates that more accurate recall for recent dates may not be a serious problem. Generalizability of these results is of concern. However, the age group that was captured was most affected in the early stages of this outbreak (*6*).

Efficient estimation of extent of disease caused by a novel infectious agent may be costly and logistically difficult. When carefully deployed, unstructured supplementary service data surveys may be a practical, low-cost, and timely complement to traditional surveillance. Further refinements of this tool are required to improve its validity. To limit recall errors and increase response rate, repeated surveys at short intervals and specific strategies to improve response rate should be considered.

## Supplementary Material

Technical Appendix Pandemic (H1N1) 2009 mobile phone unstructured supplementary service data survey questions, Characteristics of respondents and ILI cases from a mobile messaging technology survey and Reported number of nonsevere and severe cases of influenza-like illness.
