# Supplementary material for: Mobile Messaging as Surveillance Tool during Pandemic (H1N1) 2009, Mexico
Source: Emerg Infect Dis. 2010 Sep;16(9):1488–9. doi: 10.3201/eid1609.100671 (PMC3294993; doi:10.3201/eid1609.100671)
Supplement: Technical Appendix — Pandemic (H1N1) 2009 mobile phone unstructured supplementary service data survey questions, Characteristics of respondents and ILI cases from a mobile messaging technology survey and Reported number of nonsevere and severe cases of influenza-like illness. [file 10-0671-Techapp_2p.pdf]

Article DOI: 10.3201/eid1609.100671

Mobile Messaging as Surveillance Tool during Pandemic (H1N1) 2009, Mexico

Technical Appendix

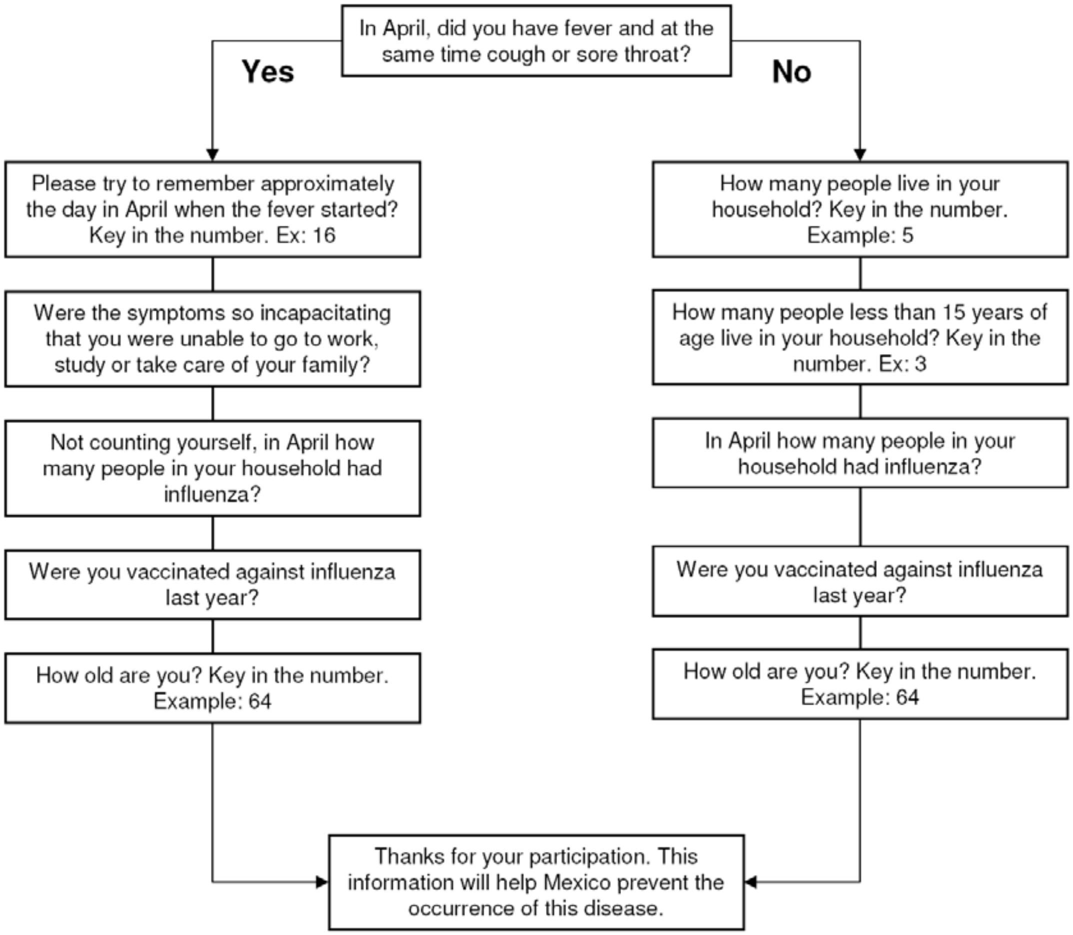

Figure 1. Pandemic (H1N1) 2009 mobile phone unstructured supplementary service data survey questions, Mexico, 2009. Ex, example.

Table. Characteristics of respondents and ILI cases from a mobile messaging technology survey, Mexico, 2009*

Characteristic	All respondents (n = 56,551)	Cases (n = 9,333)		
		All	Nonsevere (n = 4,735)	Severe (n = 4,598)
Age, y	24.7 (10.5)	23.6 (10.5)	23.5 (10)	23.8(11.2)
Severe disease, %	NA	49.3	NA	NA
No. who received influenza vaccine, %	25.9	26	26.1	25.9
No. persons with ILI in household	0.3 (1.0)	1.2 (1.9)	0.8 (1.5)	1.6 (2.1)
Location, %				
Mexico City	35.8	39.1	39.9	38.4
Nuevo Leon	17.5	12.6	13.4	11.9
San Luis Potosi	11	11.9	11.7	12.1
Quintana Roo	2.5	2.2	2.3	2.1
Rest of Mexico	32.7	33.9	34.2	33
Household size†	4.9 (2.2)	NA	NA	NA
No. persons <15 y of age in household†	1.7 (1.4)	NA	NA	NA

*Results are mean (SD) unless otherwise indicated. ILI, influenza-like illness; NA, not applicable. Missing or implausible values: age (n = 1,447) and influenza vaccination (n = 45), and persons with ILI in household (n = 329).
†Available only for noncases.

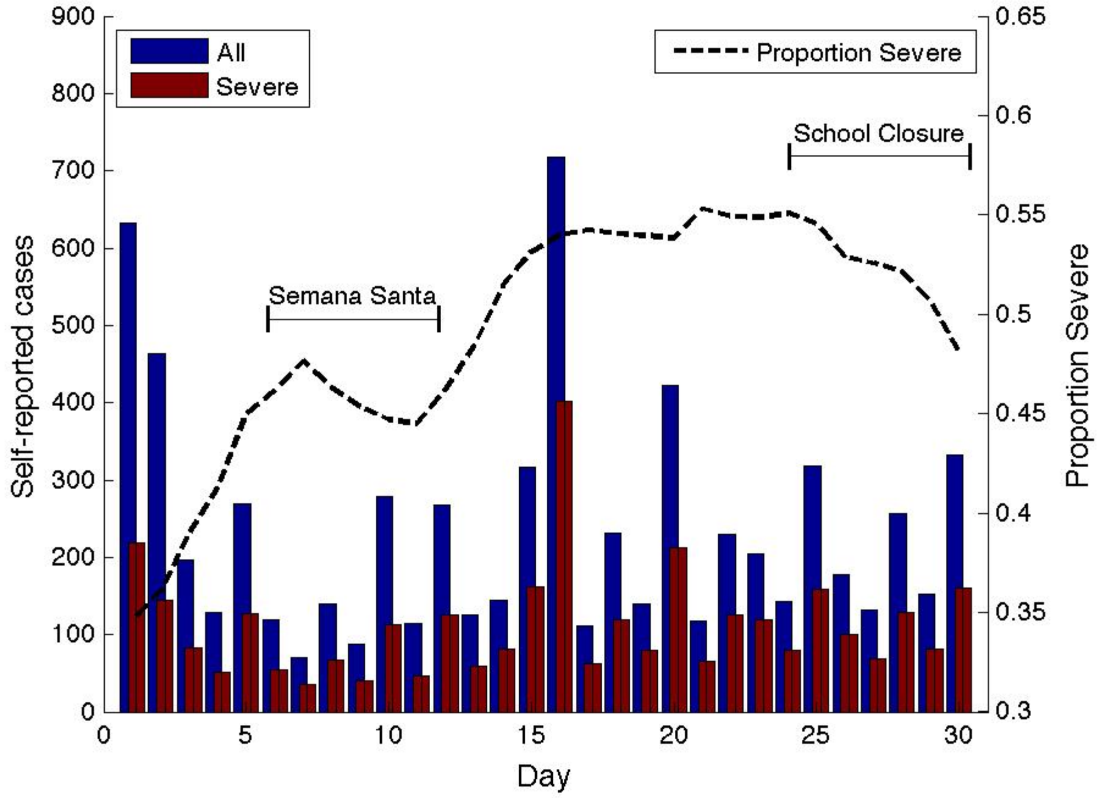

Figure 2. Reported number of nonsevere and severe cases of influenza-like illness in Mexico, April 2009, from unstructured supplementary service data survey and proportion of severe disease relative to total disease. Daily proportion of reported severe cases was smoothed by using a 5-day moving average.
